# Spirobisnaphthalenes from the Mangrove-Derived Fungus *Rhytidhysteron* sp. AS21B

**DOI:** 10.3390/md12031271

**Published:** 2014-03-06

**Authors:** Khanitha Pudhom, Thapong Teerawatananond, Supichar Chookpaiboon

**Affiliations:** 1 Department of Chemistry, Faculty of Science, Chulalongkorn University, Bangkok 10330, Thailand; E-Mail: thapong_sthc@hotmail.com; 2 Program in Biotechnology, Faculty of Science, Chulalongkorn Universtiy, Bangkok 10330, Thailand; E-Mail: bubu_keaw@hotmail.com

**Keywords:** endophytic fungus, *Rhytidhysteron* sp., spirobisnaphthalene, cytotoxicity

## Abstract

Three new spirobisnaphthalenes (**1**–**3**) were isolated from the mangrove-derived fungus *Rhytidhysteron* sp., together with five known derivatives (**4**–**8**). The structures of the compounds were established on the basis of extensive spectroscopic data, and the relative configurations of their stereogenic carbons were determined by a single-crystal X-ray crystallographic analysis. Compounds **3**–**5** displayed cytotoxicity against both cancer cell lines, MCF-7 and CaSki, while **2** was active only on CaSKi cells.

## 1. Introduction

Spirobisnaphthalenes, a series of compounds consisting of two naphthalene-derived C_10_ units bridged through a spiroketal linkage, have been mainly isolated from fungi [1,2,3,4,5]. This class of compounds is of great interest as potential leads for medicinal chemistry, since they have interesting structures and a variety of biological activities such as antibacterial, antifungal, anticancer, and antileishmanial activities [4,5,6,7].

Endophytic fungi are known as a prolific source for the discovery of structurally interesting and biologically active metabolites [8,9,10,11]. Among plant-derived fungi, those associated with the trees growing up in mangrove areas have received much attention from medicinal chemists owing to the unique ecosystem [12]. In our continued investigation into new bioactive compounds from Thai mangrove-derived fungi, we describe the isolation and structure elucidation of three new spirobisnaphthalenes, rhytidones A–C (**1**‒**3**), together with five known derivatives from an endophytic *Rhytidhysteron* sp. fungus. In addition, all isolated compounds were evaluated for their cytotoxic activities against human cancer cell lines.

## 2. Results and Discussion

**Figure 1:**
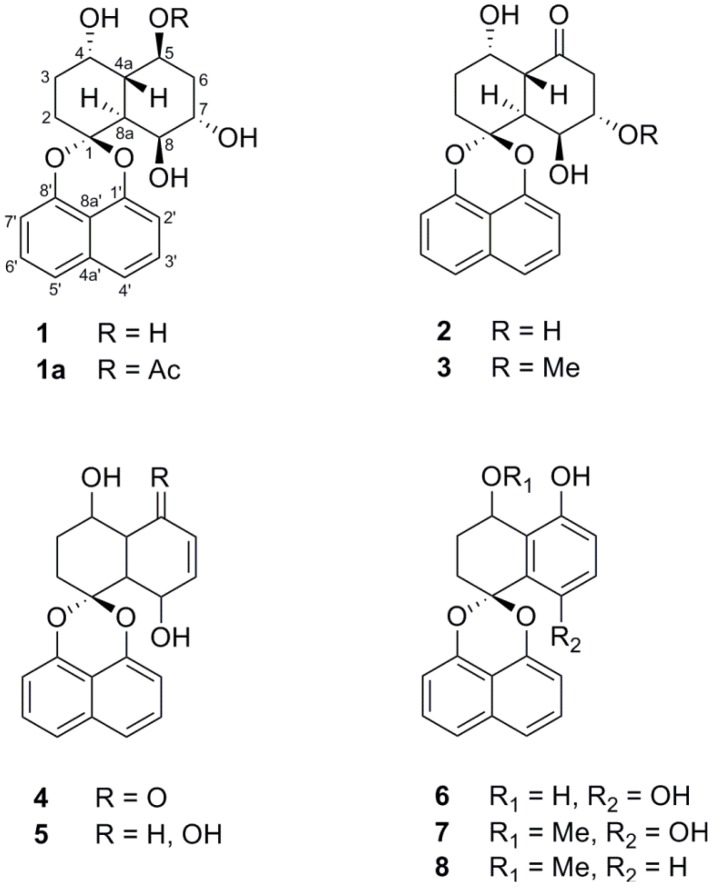
Structures of compounds **1**‒**8** isolated from fungus *Rhytidhysteron* sp.

The *Rhytidhysteron* sp. fungus was cultured in malt extract broth (MEB) under static conditions for 21 days. The EtOAc crude extract of the culture broth was successively subjected to Sephadex LH-20 and silica gel column chromatography to afford three new spironaphthalenes, rhytidones A–C (**1**–**3**), and five known analogues including MK3018 (**4**), palmarumycin CR1 (**5**), CJ-12,372 (**6**), 4-*O*-methyl-CJ-12,372 (**7**) and 4-*O*-methyl-CJ-12,371 (**8**) as shown in Figure 1. The structures of the known compounds were determined by comparison of their NMR spectroscopic data with those in the literature [13,14,15,16].

**Figure 2:**
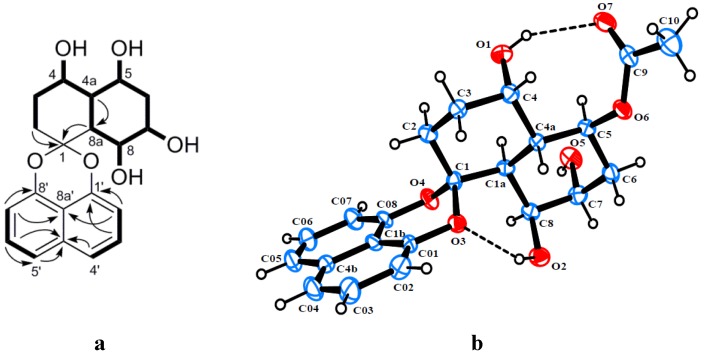
(**a**) Key HMBC and ^1^H-^1^H COSY correlations of rhytidone A (**1**); (**b**) ORTEP diagram of the 5-acetate derivative of **1** (**1a**).

Rhytidone A (**1**) was obtained as a light brown powder and its molecular formula was established as C_20_H_22_O_6_ from HRESIMS at *m/z* 381.1319 [M + Na]^+^ (calcd 381.1314), implying 10 degrees of unsaturation. Detailed analysis of the ^1^H, ^13^C and HSQC NMR data revealed the presence of six methine carbons (four oxygenated), three methylene carbons, one doubly oxygenated quaternary carbon (*δ*_C_ 104.4) and 10 aromatic carbons. In the ^1^H-^1^H COSY spectrum, homonuclear coupling correlations of H-2′/H-3′ and H-3′/H-4′, as well as correlations of H-5′/H-6′ and H-6′/H-7′ indicated the presence of two isolated three-proton spin systems corresponding to the C-2′–C-4′ and C-5′–C-7′ subunits of **1**, which displayed the *ortho* coupling constant with *J* values of 7.2, 7.6 and 8.0 Hz. The HMBC correlations of H-2′/C-1′, H-2′/C-8a′, H-3′/C-4a′, H-6′/C-4a′, H-7′/C-8′ and H-7′/C-8a′ led to the attachment of both subunits at C-4a′ and C-8a′, suggesting the presence of a naphthalene moiety. In addition, the chemical shifts of the nonprotonated carbons C-1′ and C-8′ at *δ*_C_ 147.4 and 145.9, respectively, were indicative of a 1,8-dioxynaphthalene moiety. Based on the above evidence, compound **1** was recognized as a member of the spirobisnaphthalene, characteristic of a 1,8-dioxynaphthalene linked with the second half of the molecule via a spiroketal carbon. The remaining part of the molecule was mainly elucidated by analysis of ^1^H-^1^H COSY and HMBC data. The COSY correlations confirmed the presence of the new extended spin system corresponding to the C-2–C-8 subunit (including C-4a and C-8a) (Figure 2a). HMBC correlations of H-2 and H-8a with C-1 led to the attachment of the spiroketal bridge carbon to the last subunit at C-2 and C-8a. Moreover, four exchangeable protons, observed at *δ*_H_ 4.41, 4.81, 3.85, 4.22, were assigned to OH-4, OH-5, OH-7 and OH-8, respectively, by their COSY correlations with their vicinal protons (Figure 2a). The relative configuration of **1** was determined through single-crystal X-ray diffraction analysis of its 5-acetate derivative (**1a**), obtained from acetylation of **1** with acetic anhydride in the presence of a catalytic amount of DMAP. The perspective ORTEP plot of **1a** is shown in Figure 2b.

**Figure 3:**
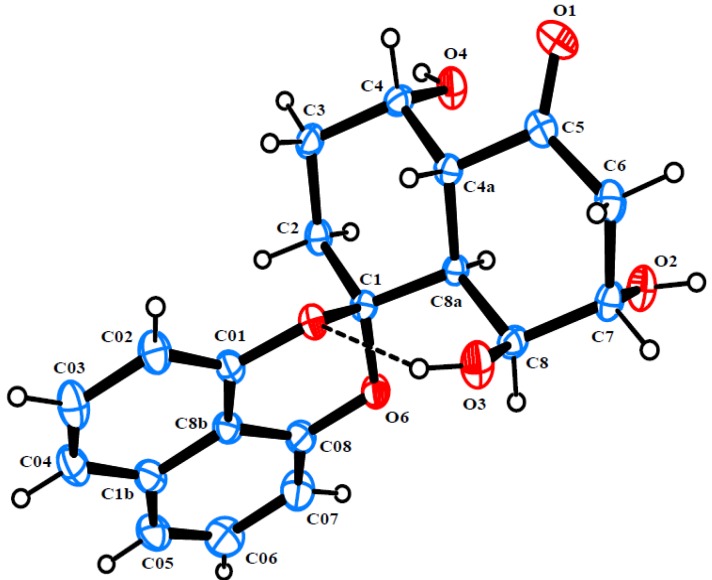
ORTEP diagram of rhytidone B (**2**).

Rhytidone B (**2**) was isolated as colorless crystals. Its HRESIMS spectrum exhibited a pseudo-molecular ion at *m/z* 379.1153 ([M + Na]^+^, calcd 379.1158), consistent with the molecular formula C_20_H_20_O_6_. The NMR data of **2** also displayed characteristic signals associated with a spirobisnaphthalene, including a 1,8-dioxynaphthalene moiety and a spiroketal bridge carbon. Moreover, its NMR data (Table 1) were similar to those of **1**, except for the replacement of one oxygenated methine carbon in **1** by a new ketone carbon (*δ*_C_ 212.2). The –CH_2_(2)-CH_2_(3)-CH(4)OH-CH(4a)-CH(8a)-CH(8)OH-CH(7)-OH-CH_2_(6)– subunit was established by analysis of ^1^H-^1^H COSY. HMBC correlations from H-4a and H_2_-6 to the ketone carbon clearly indicated the location of the ketone carbonyl at C-5. Ultimately, the structure and relative configuration of **2** was clarified by single-crystal X-ray diffraction analysis (Figure 3).

**Table 1:** NMR spectroscopic data of compounds **1**–**3**.

No.	1 ^a^		2 ^b^		3 ^b^	
	*δ*_H_ (*J* in Hz)	*δ*_C_	*δ*_H_ (*J* in Hz)	*δ*_C_	*δ*_H_ (*J* in Hz)	*δ*_C_
1	-	104.4	-	104.4	-	104.4
2	1.73, m	25.6	1.95, m	25.6	1.94, m	25.6
3	1.53, m	28.2	1.75, m 1.66, m	26.5	1.72, m 1.62, m	26.6
4	4.16, br s	61.2	4.54, br s	62.9	4.52, br s	62.9
4a	1.97, ddd (12.8, 10.0, 2.4)	42.9	3.31, d (13.6)	49.0	3.27, dd (13.2, 1.6)	48.9
5	3.70, br s	68.8	-	212.2	-	211.8
6	1.75, m	35.7	3.13, br s 2.47, dd (14.4, 2.8)	44.2	3.05, m 2.60, dd (14.4, 2.0)	41.2
7	4.21, br s	66.9	4.43, t (3.2)	71.3	3.89, m	80.1
8	3.93, m	63.3	4.77, d (3.2)	67.4	4.88, br d (3.6)	65.1
8a	2.44, dd (12.8, 1.6)	38.6	3.17, br s	41.5	3.03, m	41.9
1'	-	146.5	-	147.3	-	-
2'	6.96, d (7.2)	109.4	6.94, d (7.2)	109.8	6.96, d (7.6)	109.8
3'	7.45, t (7.6)	127.6	7.43, t (8.0)	127.8	7.44, t (7.6)	127.7
4'	7.50, d (8.0)	119.7	7.53, d (8.4)	121.5	7.48, d (8.4)	120.5
4a'	-	133.6	-	134.2	-	134.2
5'	7.52, d (8.0)	120.1	7.49, d (8.4)	120.5	7.53, d (8.4)	121.4
6'	7.45, t (7.6)	127.5	7.43, t (8.0)	127.1	7.42, t (7.6)	127.1
7'	6.94, d (7.2)	108.8	6.95, d (7.2)	109.6	6.93, d (7.6)	109.6
8'	-	147.6	-	145.9	-	145.9
8a'	-	113.3	-	113.8	-	113.9
4-OH	3.70, br s	-	-	-	-	-
5-OH	4.81, d (2.8)	-	-	-	-	-
7-OH	3.85, d (2.4)	-	-	-	-	-
8-OH	4.21, br s	-	3.77, s	-	3.71, s	-
7-OMe	-	-	-	-	3.43, s	56.9

^a^ Measured in DMSO-d_6_; ^b^ measured in CDCl_3_.

**Figure 4:**
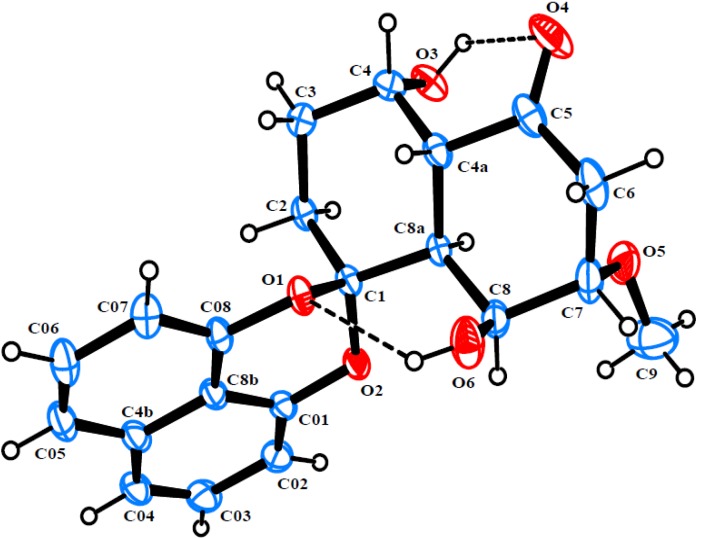
ORTEP diagram of rhytidone C (**3**).

Rhytidone C (**3**), obtained as colorless crystals, gave the molecular formula C_21_H_22_O_6_, as established by HRESIMS (*m/z* 393.1315 ([M + Na]^+^, calcd. 393.1314). The NMR data of **3** (Table 1) were very similar to those of 2, except for the presence of an additional methoxy group (*δ*_H_ 3.43 s, *δ*_C_ 56.9). Strong HMBC correlation from methoxyl protons to C-7 at *δ*_C_ 80.1 indicated the attachment of the methoxyl group at C-7. Compound **3** was found to have the same configuration as in **2**, which was also determined by single-crystal X-ray diffraction analysis (Figure 4).

Cytotoxic activity of isolated compounds against human breast cancer (MCF-7) and cervical carcinoma (CaSki) cell lines was evaluated by the MTT method (Table 2) [17]. Compounds **3**‒**5** showed moderate activity against both cell lines with IC_50_ values between 14.47 and 25.59 μM, while compound **2** was active only for CaSki with IC_50_ value of 22.81 μM. It should be noted that compounds **6**‒**8**, possessing an additional aromatic ring in the structure, did not exhibit any significant activity against the cell lines tested (IC_50_ > 10 μg/mL).

More details are available at the Supplementary Information.

**Table 2:** Cytotoxicity of compounds **2**–**5** against human breast and cervical cancer cell lines.

Compound	IC_50_ (μM)	
	MCF-7	CaSki
**2**	− ^a^	22.81 ± 1.33
**3**	17.30 ± 2.11	24.44 ± 0.22
**4**	20.10 ± 1.52	25.59 ± 1.70
**5**	14.47 ± 0.51	21.95 ± 2.56
Doxorubicin	0.06 ± 0.01	0.20 ± 0.02

^a^ IC_50_ > 10 μg/mL.

## 3. Experimental Section

### 3.1. General Experimental Procedures

Optical rotations were measured on a Perkin-Elmer 341 polarimeter. UV spectra were recorded on a Shimadzu UV-160 UV-visible spectrometer (Shimadzu, Kyoto, Japan). NMR spectra were acquired on a Varian Mercury-400 Plus NMR spectrometer (Varian, CA, USA) with TMS as internal standard. HRESIMS was carried out on a micrOTOF-Q II ESI mass spectrometer (Bruker, Bremen, Germany). Single-crystal X-ray diffraction analysis was performed on a Bruker APEX II diffractometer (Bruker, Bremen, Germany).

### 3.2. Fungal Material and Fermentation

The fungus AS21B used in the present study was isolated from leaves of *Azima sarmentosa*, collected from the mangrove forest in Samutsakhon province, Thailand in July 2008. The fungus was identified as a *Rhytidhysteron* sp. based on the ITS sequences, and was deposited at Department of Chemistry, Faculty of Science, Chulalongkorn Universtiy. The strain AS21B was grown on potato dextrose agar (PDA) plate at room temperature for 7 days. Five pieces (5 × 5 mm^2^) of mycelial agar plugs were inoculated into 1 L Erlenmeyer flasks (×50) containing 200 mL of malt extract broth (MEB). The cultivation was kept at room temperature for 21 days under static conditions.

### 3.3. Extraction and Isolation

The mycelia were separated off from the broth by filtration. The filtrate was extracted with an equal amount of EtOAc for 3 times. The EtOAc solution was evaporated under reduced pressure to afford a crude extract (7.0 g). The extract was subjected to a Sephadex LH20 column and eluted with MeOH to give six fractions (F1–F6). Subsequently, fraction 5 was fractionated by silica gel (SiO_2_) column chromatography eluted by a gradient of MeOH/CH_2_Cl_2_ from 1:99 to 1:9 to yield nine subfractions. The fraction F5.3 was purified by SiO_2_ column chromatography (a gradient of EtOAc/hexane from 2:8 to 1:1) to give **3** (20.1 mg) and **4** (47.8 mg). Subfraction F5.4 was rechromatographed on SiO_2_ with a 1:1 mixture of EtOAc/hexane to obtain **2** (13.5 mg). Fraction F5.6 was applied to SiO_2_ column chromatography eluted with MeOH/CH_2_Cl_2_ (1:19) to afford 5 (15.2 mg). Fraction F5.7 was divided into four fractions by column chromatography on Sephadex LH20 (MeOH), then F5.7.4 was further purified by SiO_2_ column chromatography with MeOH/CH_2_Cl_2_ (1:9) to give **1** (83 mg). Fraction F6 was subjected to a Sephadex LH20 column eluted with MeOH to yield five subfractions. F6.4 was recrystallized with MeOH to provide **7** (32.6 mg). Subfraction F6.5 was rechromatographed over a SiO_2_ column to give three fractions. The fraction F6.5.3 was further purified by SiO_2_ column chromatography eluted with EtOAc/hexane (1:3) to yield **6** (12.8 mg) and **8** (8.2 mg).

Rhytidone A (**1**): light brown powder; [α]^25^_D_ +21 (*c* 0.10, MeOH); ^1^H and ^13^C NMR, see Table 1; HRESIMS *m/z* 381.1319 [M + Na]^+^ (calcd for C_20_H_22_O_6_Na, 381.1314).

Rhytidone B (**2**): colorless crystals; [α]^25^_D_ +15 (*c* 0.10, MeOH); ^1^H and ^13^C NMR, see Table 1; HRESIMS *m/z* 379.1153 [M + Na]^+^ (calcd for C_20_H_20_O_6_Na, 379.1158).

Rhytidone C (**3**): colorless crystals; [α]^25^_D_ +18 (*c* 0.10, MeOH); ^1^H and ^13^C NMR, see Table 1; HRESIMS *m/z* 393.1315 [M + Na]^+^ (calcd for C_21_H_22_O_6_Na, 393.1314).

Preparation of 5-*O*-Acetyl-rhytidone A (**1a**). A mixture of compound **1** (10 mg), acetic anhydride (0.1 mL) and a catalytic amount of DMAP in CH_2_Cl_2_ (2 mL) was stirred at room temperature for 30 min. Then, water (5 mL) was added to the reaction mixture, and extracted with EtOAc (3 × 5 mL). The combined organic layer was washed with brine, dried over MgSO_4_, and evaporated after filtration. The residue was purified by SiO_2_ column chromatography (MeOH/CH_2_Cl_2_, 2:98) to yield **1a** (7.2 mg, 64%) as colorless crystals: ^1^H NMR (CDCl_3_, 400 MHz) *δ* 7.51 (1H, d, *J* = 8.4 Hz, H-5′), 7.47 (1H, d, *J* = 8.4 Hz, H-4′), 7.44 (1H, t, *J* = 7.2 Hz, H-6′), 7.39 (1H, t, *J* = 7.2 Hz, H-3′), 6.93 (1H, d, *J* = 8.4 Hz, H-2′), 6.90 6.90 (1H, d, *J* = 8.4 Hz, H-7′), 5.34 (1H, ddd, *J* = 5.2, 10.0, 16.8 Hz, H-5), 4.56 (1H, br s, H-8), 4.13 (1H, br m, H-7), 3.88 (1H, br s, 4-OH), 3.70 (1H, s, 8-OH), 3.58 (1H, br s, H-4), 2.76 (1H, dd, *J* = 1.6, 12.8 Hz, H-8a), 2.43 (1H, ddd, *J* = 2.0, 12.8, 16.8 Hz, H-4a), 2.27 (1H, ddd, *J* = 2.4, 12.8, 14.4 Hz, H-6a), 2.15 (3H, s, 5-OAc), 2.00 (1H, m, H-6b), 1.97 (2H, m, H-2), 1.73 (2H, m, H-2); ^13^C NMR (CDCl_3_, 100 MHz) *δ* 172.7 (5-O*C*OCH_3_), 147.5 (C-8′), 146.0 (C-1′), 134.2 (C-4a′), 127.7 (C-6′), 127.0 (C-3′), 121.3 (C-5′), 120.3 (C-4′), 113.9 (C-8a′), 109.8 (C-2′), 109.5 (C-7′), 104.7 (C-1), 69.4 (C-5), 69.2 (C-7), 67.6 (C-8), 62.6 (C-4), 41.3 (C-4a), 39.0 (C-8a), 32.3 (C-6), 27.2 (C-3), 25.6 (C-2), 21.2 (5-OCOCH_3_).

X-ray Crystallographic Analysis of compounds **1a**, **2** and **3**. All single crystal X-ray diffraction data were collected at 296 K on a Bruker APEX II diffractometer with Mo Kα radiation (λ = 0.71073 Å). The structures were solved by direct methods using SHELXS-97 and refined full-matrix least squares on all *F*^2^ data using SHELXL97 to final *R* values [18,19]. All hydrogen atoms were added at calculated positions and refined using a rigid model. Crystallographic data for **1a**, **2** and **3** have been deposited with the Cambridge Crystallographic Data Centre (Cambridge, UK) [20].

Crystal data of **1a**: colorless crystal; C_22_H_24_O_7_, *M_r_* = 400.41, monoclinic, *a* = 12.220(2) Å, *b* = 5.7498(9) Å, *c* = 13.925(3) Å, space group *P*2_1_, *Z* = 2 and *V* = 956.2(3) Å^3^, μ(Mo Kα) = 0.10 mm^−1^, and *F*(000) = 424. Crystal dimensions: 0.42 × 0.27 × 0.25 mm. Independent reflections: 1492 (*R*_int_ = 0.063). The final *R*_1_ values were 0.039, w*R*_2_ = 0.113 (*I* > 2σ(*I*)). CCDC number: 977804.

Crystal data of **2**: colorless crystal; C_20_H_20_O_6_, *M_r_* = 356.36, orthorhombic, *a* = 7.6970(6) Å, *b* = 8.4012(8) Å, *c* = 25.845(2) Å, space group *P*2_1_2_1_2_1_, *Z* = 4 and *V* = 1617.3(2) Å^3^, μ(Mo Kα) = 0.11 mm^−1^, and *F*(000) = 752. Crystal dimensions: 0.40 × 0.22 × 0.20 mm. Independent reflections: 3200 (*R*_int_= 0.025). The final *R*_1_ values were 0.037, w*R*_2_ = 0.110 (*I* > 2σ(*I*)). CCDC number: 977805.

Crystal data of **3**: colorless crystal; C_21_H_22_O_6_, *M_r_* = 370.39, monoclinic, *a* = 23.9484(6) Å, *b* = 5.6338(1) Å, *c* = 15.5486(4) Å, space group *P*2_1_2_1_2_1_, *Z* = 4 and *V* = 1783.37(7) Å^3^, μ(Mo Kα) = 0.10 mm^−1^, and *F*(000) = 784. Crystal dimensions: 0.36 × 0.34 × 0.24 mm. Independent reflections: 3133 (*R*_int_ = 0.016). The final *R*_1_ values were 0.037, w*R*_2_ = 0.119 (*I* > 2σ(*I*)). CCDC number: 898711.

### 3.4. Cytotoxicity Assay

Cytotoxicity of all compounds was assayed with a modification of the MTT (3-[4,5-dimethylthiazol-2-yl-2,5-diphenyltetrazolium] bromide) colorimetric method. Cytotoxicity assays were performed according to previously described procedures [16]. The following human cancer cell lines were used in the assay: human breast cancer (MCF-7) and cervical carcinoma (CaSki) cell lines. Doxorubicin was used as the reference compound. 

## 4. Conclusions

The chemical investigation of the EtOAc extract of the endophytic fungus *Rhytidhysteron* sp. has led to the isolation and characterization of three spirobisnaphthalenes, along with five known derivatives. All isolated compounds (**1**‒**8**) were evaluated for their cytotoxicity against breast and cervical cancer cells. Compounds **3**‒**5** exhibited cytotoxicity against both cell lines, whereas compound **2** was active only for cervical carcinoma cells.

## Supplementary Files

Supplementary File 1 Supplementary Information (PDF, 1501 KB)

## References

[1] Jiao P., Swenson D.C., Gloer J.B., Campbell J., Shearer C.A. (2006). Decaspirones A‒E, bioactive spirodioxynaphthalenes from the freshwater aquatic fungus *Decaisnella thyridioides*. J. Nat. Prod..

[2] Hu H., Guo H., Li E., Zhou Y., Che Y. (2006). Decaspirones F‒I, bioactive secondary metabolites from the saprophytic fungus *Helicoma viridis*. J. Nat. Prod..

[3] Macías-Rubalcava M.L., Hernández-Bautista B.E., Jiménez-Estrada M., González M.C., Glenn A.E., Hanlin R.T., Hernández-Ortega S., Saucedo-García A., Muria-González J.M., Anaya A.L. (2008). Naphthaquinone spiroketal with allelochemical activity from the newly discovered endophytic fungus *Edenia gomezpompae*. Phytochemistry.

[4] Matínez-Luis S., Della-Togna G., Coley P.D., Kursar T.A., Gerwick W.H., Cubilla-Rios L. (2008). Antileishmanial constituents of the Panamanian endophytic fungus *Edenia* sp. J. Nat. Prod..

[5] Van der Sar S.A., Blunt J.W., Munro M.H.G. (2006). Spiro-Mamakone A, a unique relative of the spironaphthalene class of compounds. Org. Lett..

[6] Chen X., Shi Q., Lin G., Guo S., Yang J. (2009). Spirobisnaphthalene analogues from the endophytic fungus *Preussia* sp.. J. Nat. Prod..

[7] Cai Y.-S., Guo Y.-W., Krohn K. (2010). Structure, bioactivities, biosynthetic relationships and chemical synthesis of the spirodioxynaphthalenes. Nat. Prod. Rep..

[8] Zhang H.W., Song Y.C., Tan R.X. (2006). Biology and chemistry of endophytes. Nat. Prod. Res..

[9] Gunatilaka A.A.L. (2006). Natural products from plant-associated microorganisms: Distribution, structural diversity, bioactivity and implications of their occurrence. J. Nat. Prod..

[10] Strobel G., Diasy B., Castillo U., Harper J.  (2004). Natural products form endophytic microorganisms. J. Nat. Prod..

[11] Strobel G.A. (2003). Endophytes as sources of bioactive products. Microbes Infect..

[12] Debbab A., Aly A.H., Proksch P. (2013). Mangrove derived fungal endophytes—A chemical and biological perception. Fungal Divers..

[13] Sakemi S., Inagaki T., Kaneda K., Hirai H., Iwata E., Sakakibara T., Yamauchi Y., Norcia M., Wondrack L.M., Sutcliffe J.A., Kojima N. (1995). CJ-12,371 and CJ-12,372, two novel DNA gyrase inhibitors, fermentation, isolation, structural elucidation and biological activities. J. Antibiot..

[14] Wipf P., Lynch S.M., Birmingham A., Tamayo G., Jiménez A., Campos N., Powis G.  (2004). Natural product based inhibitors of the thioredoxin-thioredoxin reductase system. Org. Biomol. Chem..

[15] Ragot J.P., Steeneck C., Alcaraz M.-L., Taylor R.J.K. (1999). The synthesis of 1,8-dihydroxynaphthalene-derived natural products: Palmarymycin CP_1_, Palmarymycin CP_2_, Palmarymycin CP_11_, CJ-12,371, deoxypreussomerin A and novel analogues. J. Chem. Soc. Perkin Trans. 1.

[16] Ohishi H., Chiba N., Mikawa T., Sakaki T., Miyaji S., Sezaki M.  (1989). Novel Antibiotic MK3018 substance— Useful as antimicrobial agent. Jpn. Pat..

[17] Chokpaiboon S., Sommit D., Teerawatananond T., Muangsin N., Bunyapaiboonsri T., Pudhom K. (2010). Cytotoxic nor-chamigrane and chamigrane endoperoxides from a basidiomycetous fungus. J. Nat. Prod..

[18] Sheldrick G.M. (1997). SHELXS-97, Program for Crystal Structure Solution.

[19] Sheldrick G.M. (1997). SHELXS-97, Program for Crystal Structure Refinement.

[20] Cambridge Crystallographic Data Centre. http://www.ccdc.cam.ac.uk/data_request/cif.

