# Supplementary material for: Spirobisnaphthalenes from the Mangrove-Derived Fungus Rhytidhysteron sp. AS21B
Source: Mar Drugs. 2014 Mar 6;12(3):1271–80. doi: 10.3390/md12031271 (PMC3967209; doi:10.3390/md12031271)
Supplement: Supplementary File 1 — Supplementary Information (PDF, 1501 KB) [file marinedrugs-12-01271-s001.pdf]

Supplementary Information

Figure S1. ^{1}H NMR Spectrum of Rhytidone A (**1**) in DMSO-d_6.

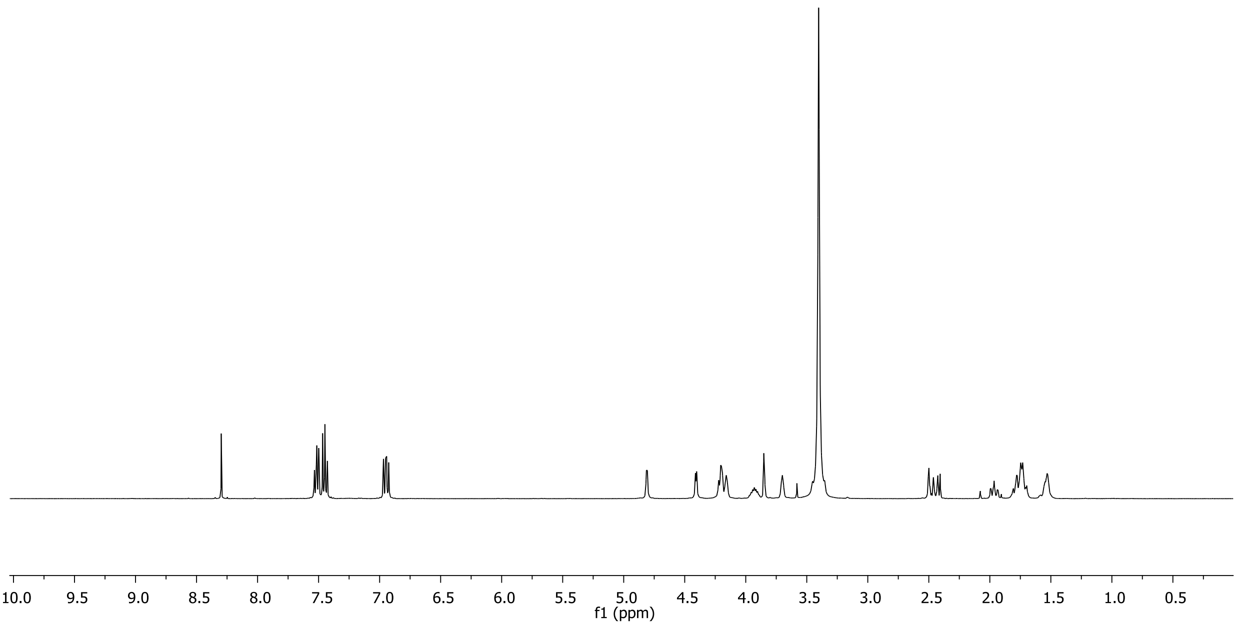

Figure S2. ^{13}C NMR Spectrum of Rhytidone A (**1**) in DMSO-d_6.

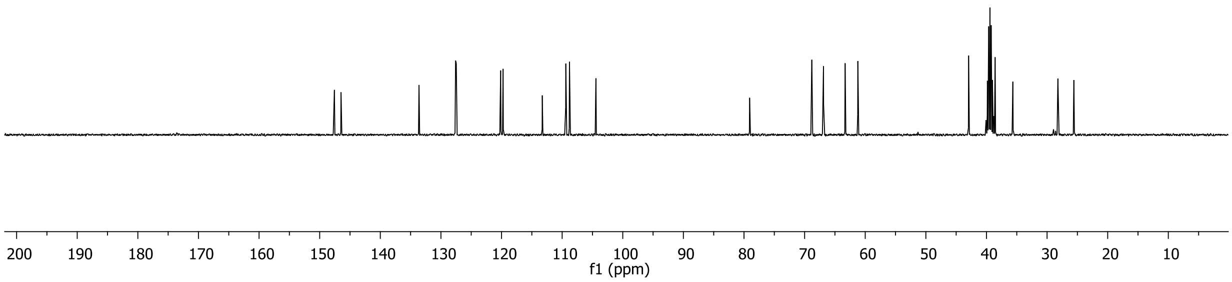

Figure S3. ^{1}H-^{1}H COSY Spectrum of Rhytidone A (**1**) in DMSO-d_6.

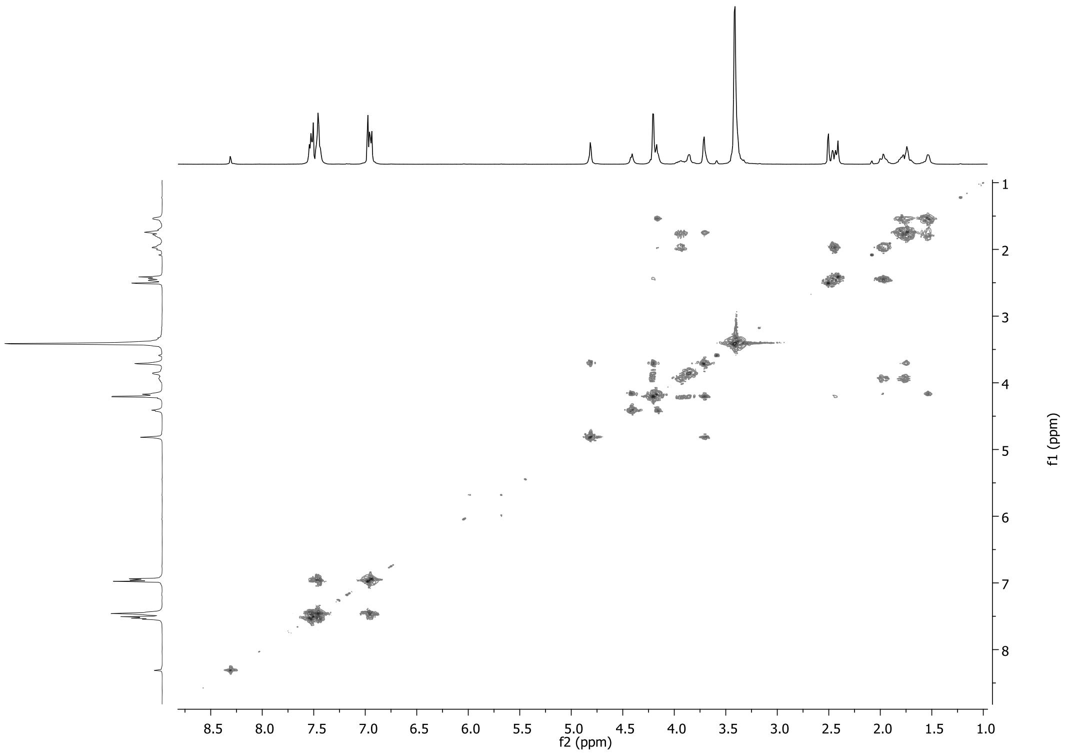

Figure S4. HSQC Spectrum of Rhytidone A (**1**) in DMSO-d_6.

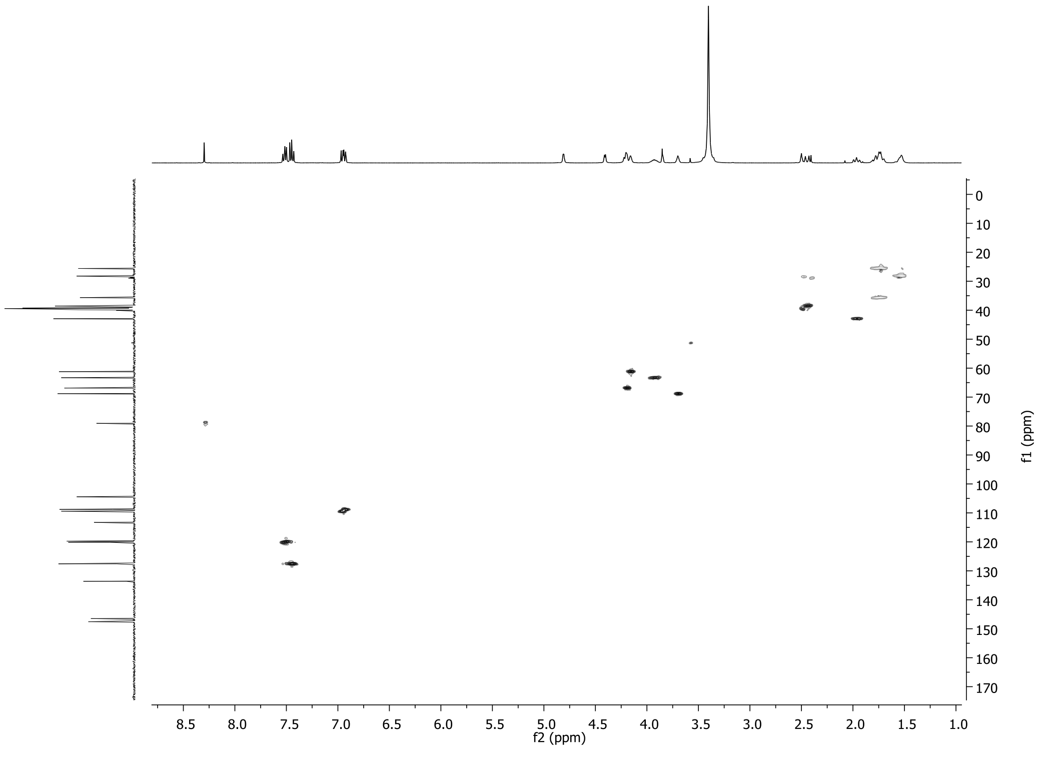

Figure S5. HMBC Spectrum of Rhytidone A (**1**) in DMSO-d_6.

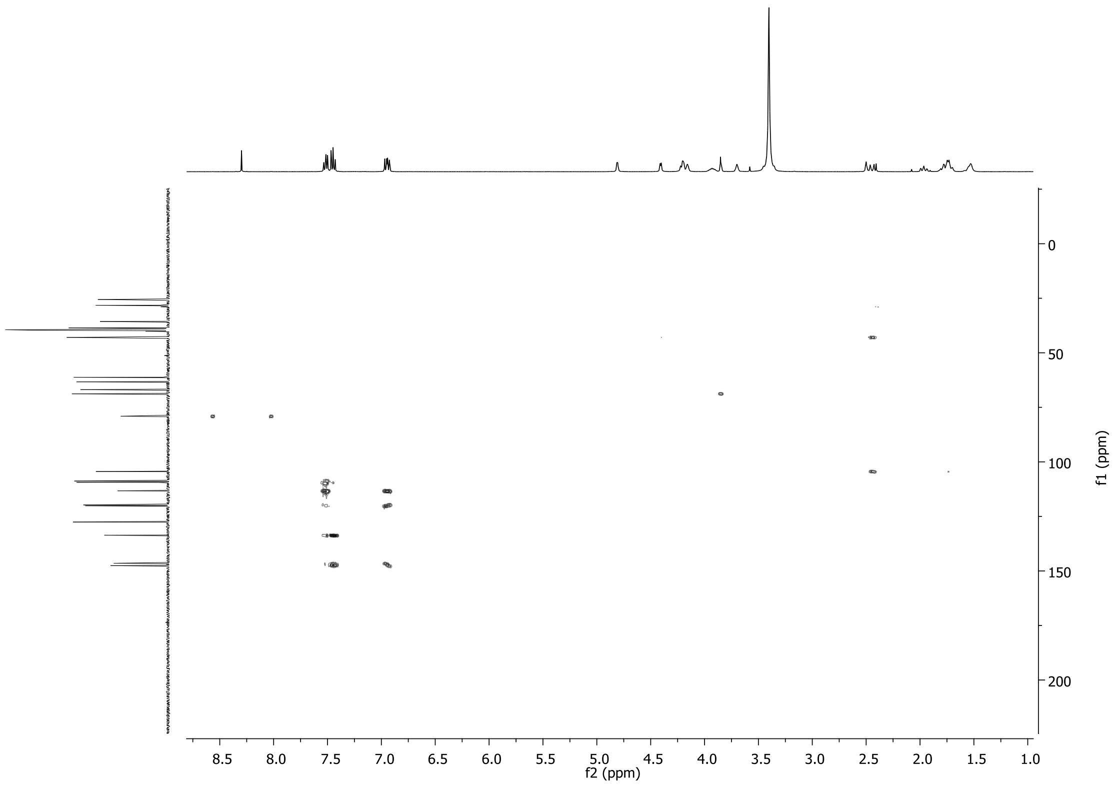

Figure S6. ^{1}H NMR Spectrum of Compound **1a** in $CDCl_3$.

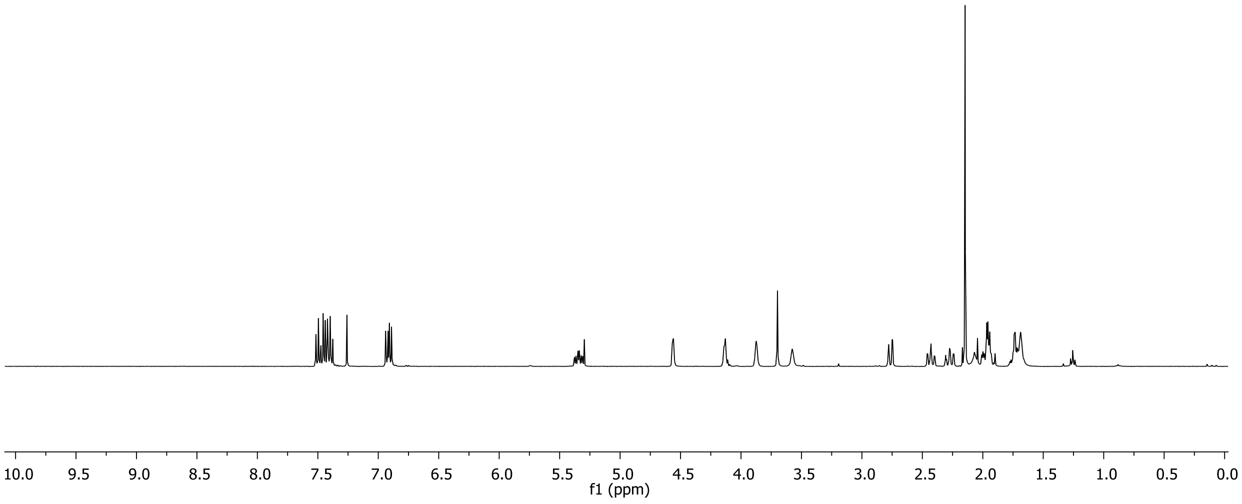

Figure S7. ^{13}C NMR Spectrum of Compound **1a** in $CDCl_3$.

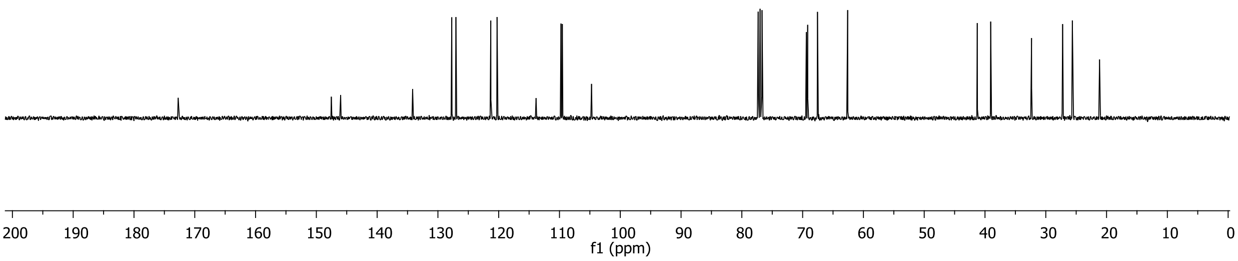

Figure S8. ^{1}H-^{1}H COSY Spectrum of Compound **1a** in $CDCl_3$.

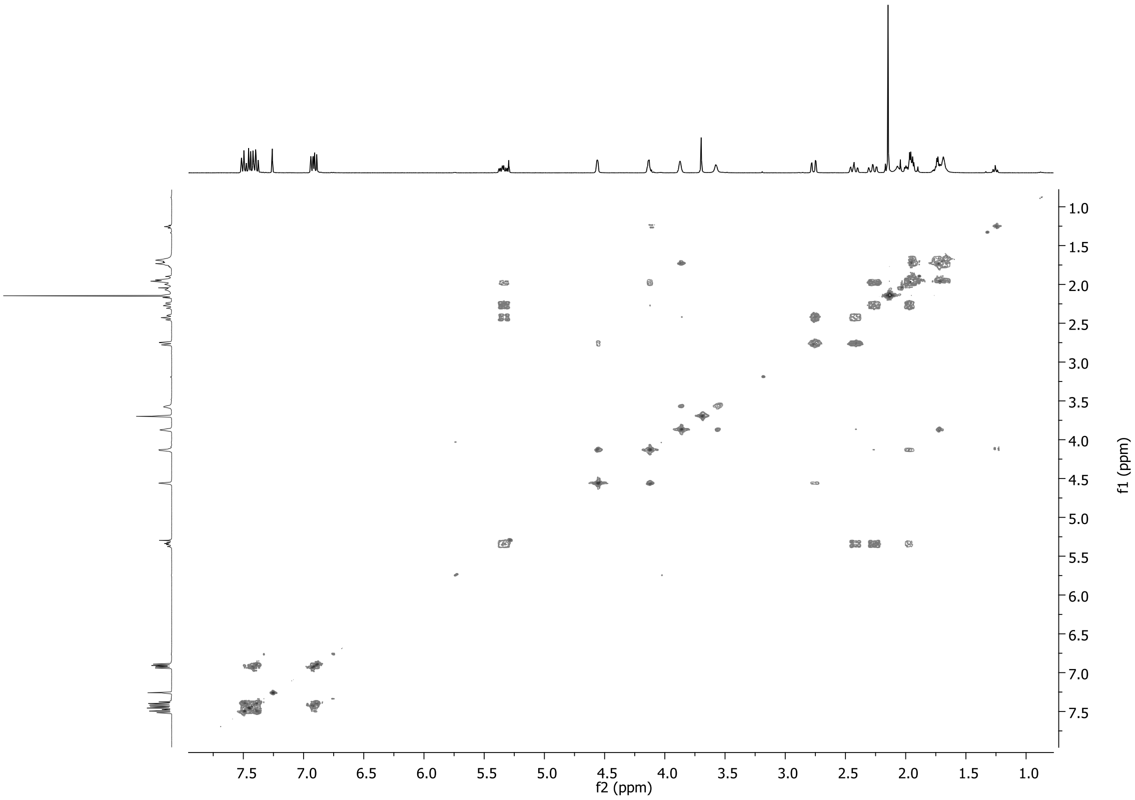

Figure S9. HSQC Spectrum of Compound **1a** in $CDCl_3$.

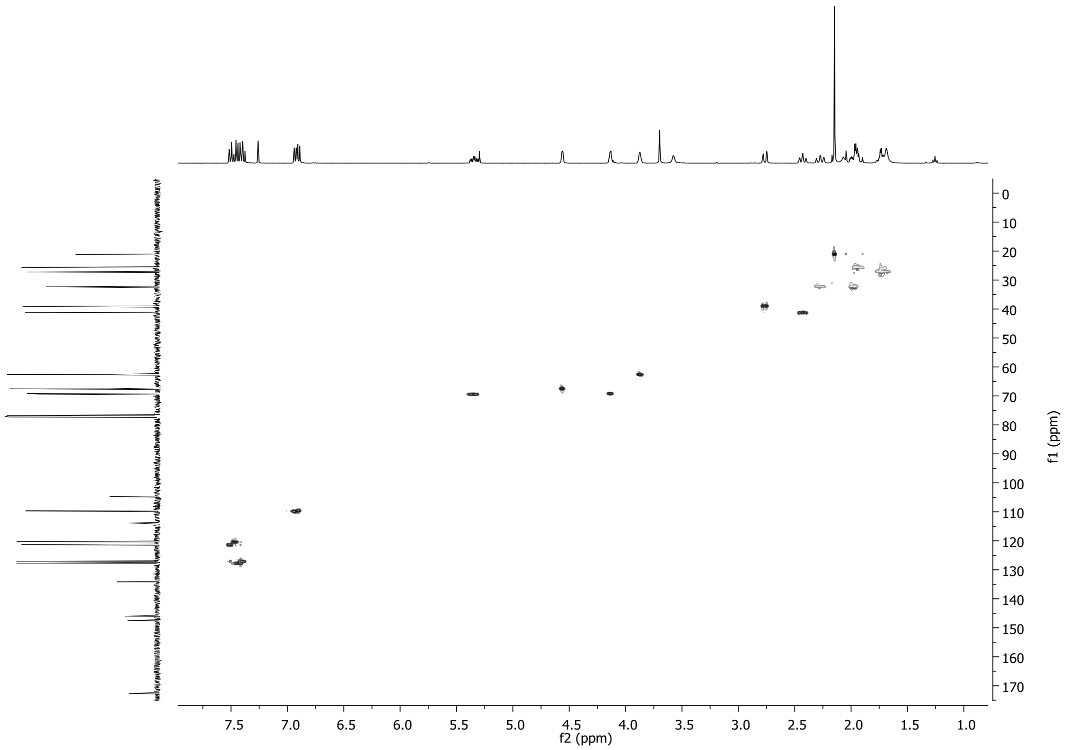

Figure S10. HMBC Spectrum of Compound **1a** in $CDCl_3$.

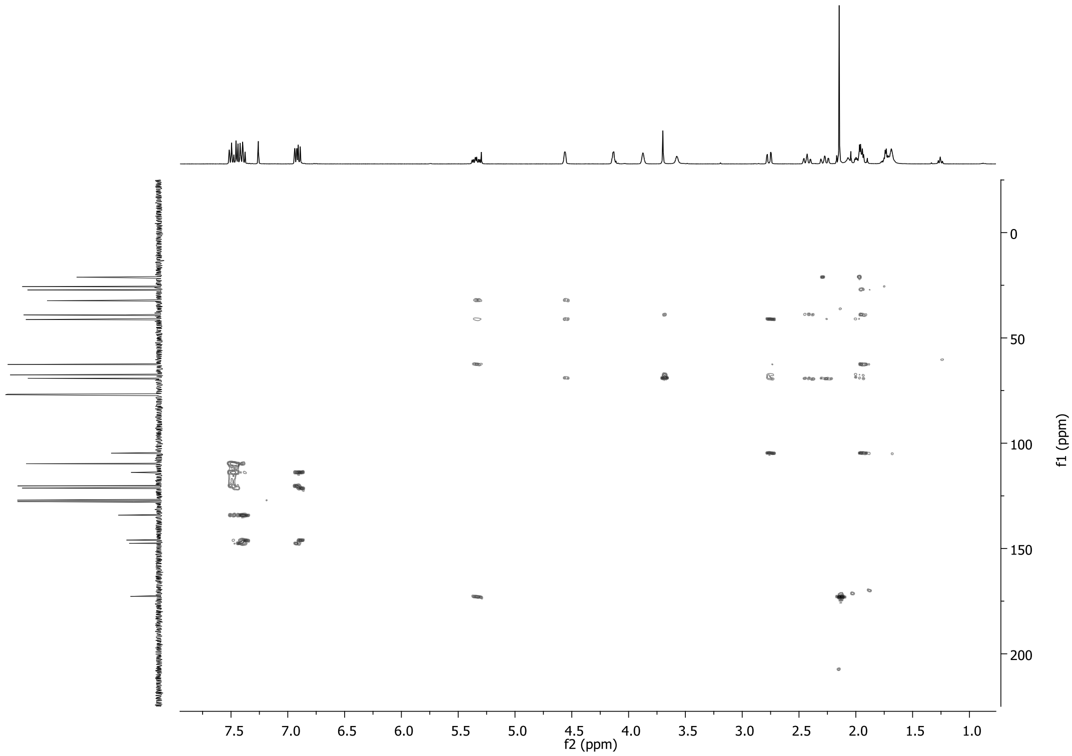

Figure S11. ^{1}H NMR Spectrum of Rhytidone B (**2**) in $CDCl_3$.

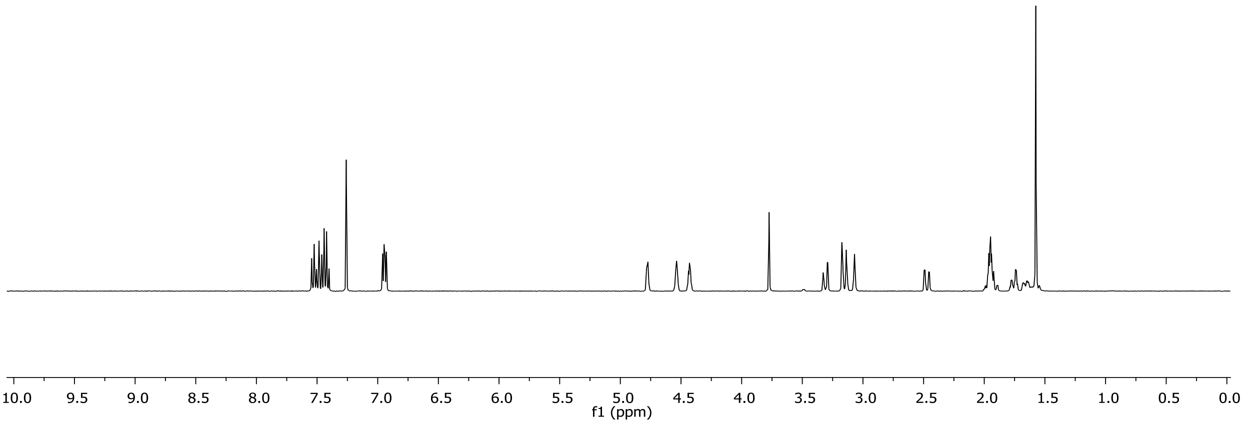

Figure S12. ^{13}C NMR Spectrum of Rhytidone B (**2**) in $CDCl_3$.

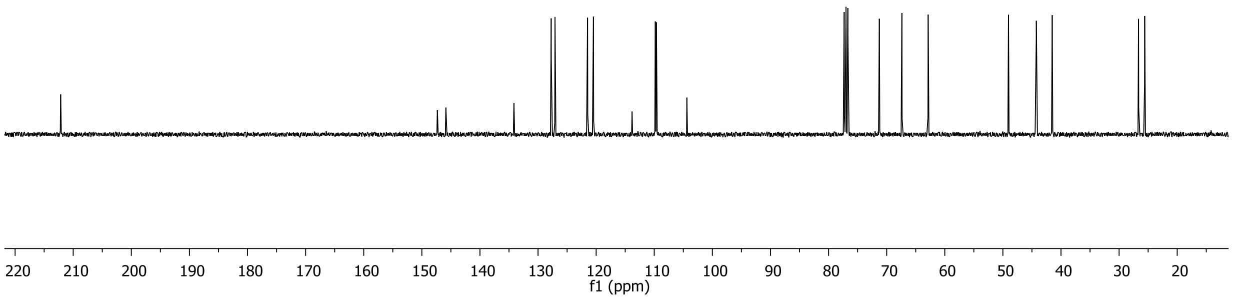

Figure S13. ^{1}H-^{1}H COSY Spectrum of Rhytidone B (**2**) in $CDCl_3$.

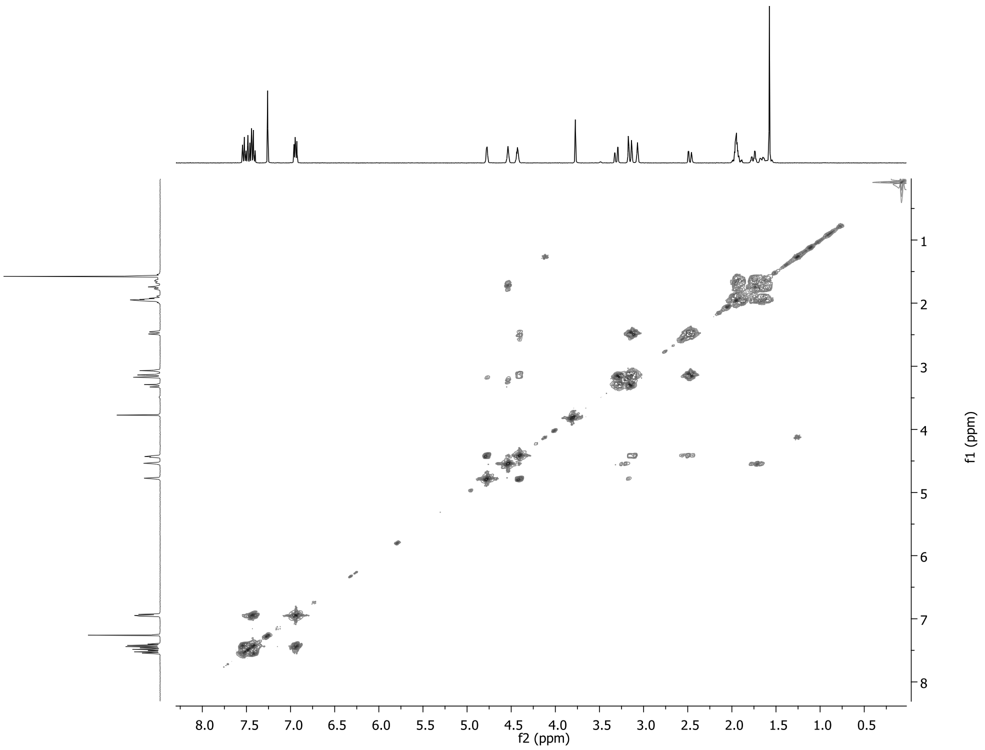

Figure S14. HSQC Spectrum of Rhytidone B (**2**) in $CDCl_3$.

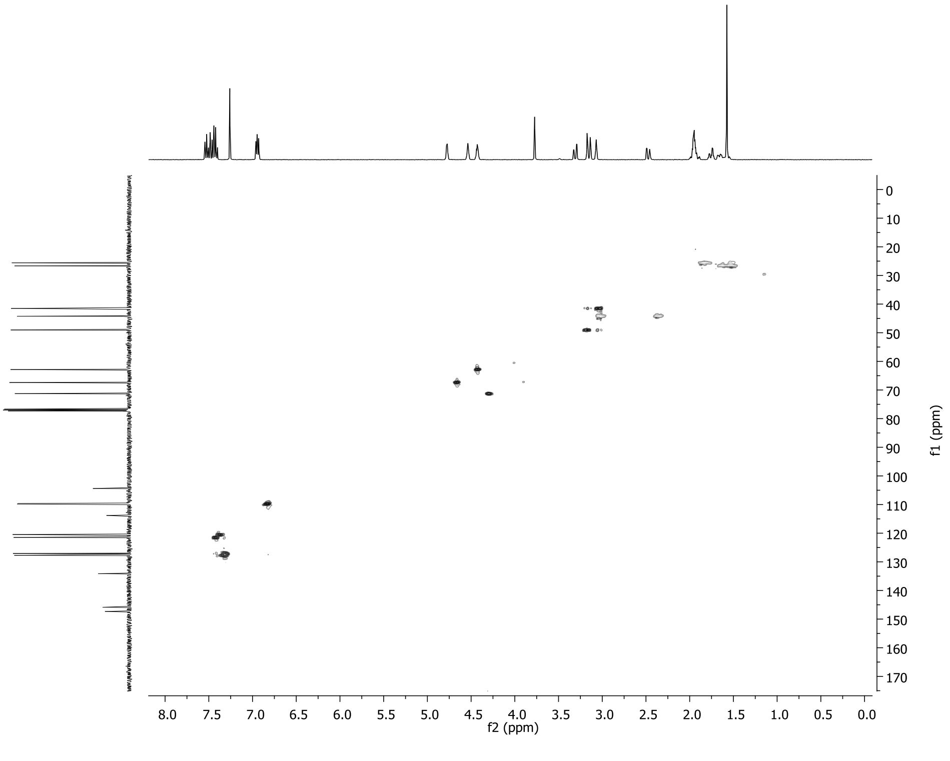

Figure S15. HMBC Spectrum of Rhytidone B (**2**) in $CDCl_3$.

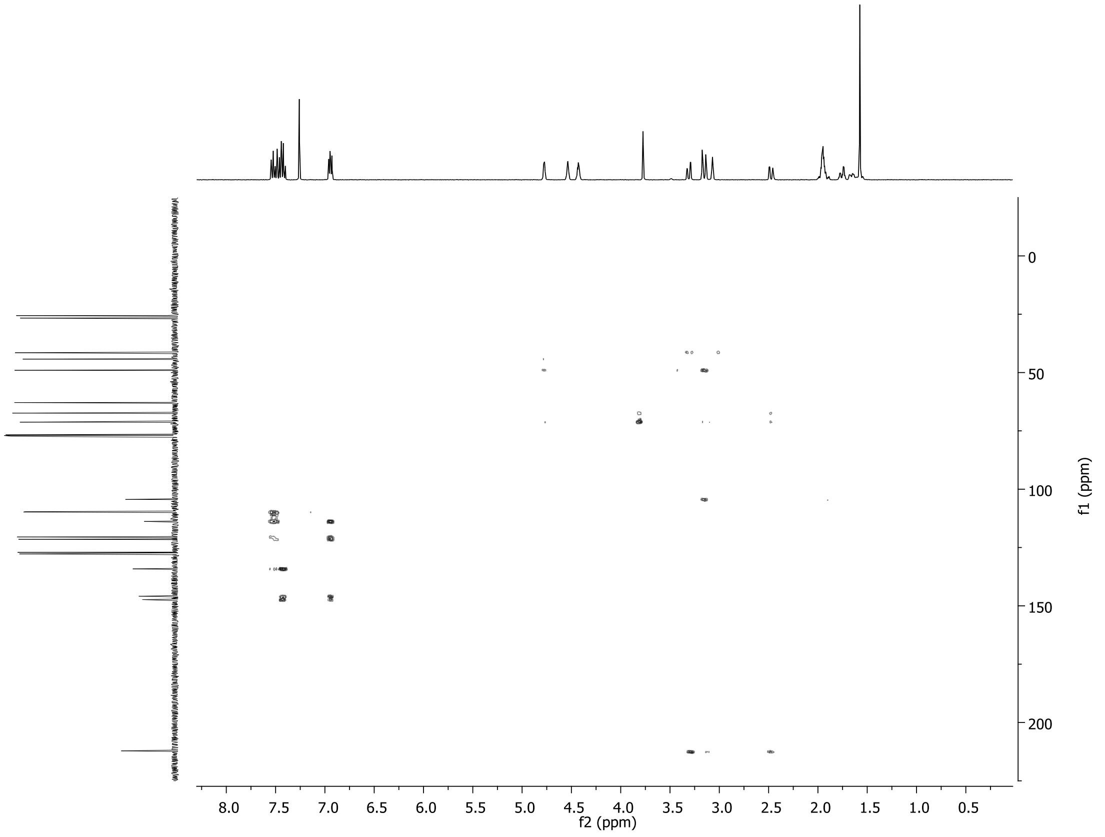

Figure S16. ^{1}H NMR Spectrum of Rhytidone C (**3**) in $CDCl_3$.

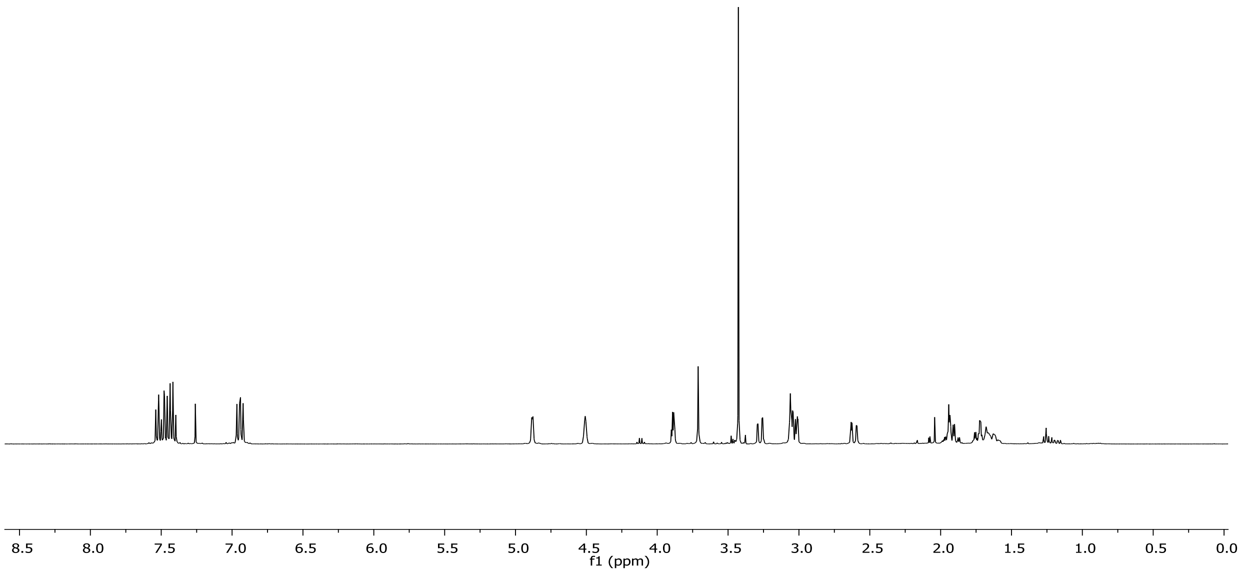

Figure S17. ^{13}C NMR Spectrum of Rhytidone C (**3**) in $CDCl_3$.

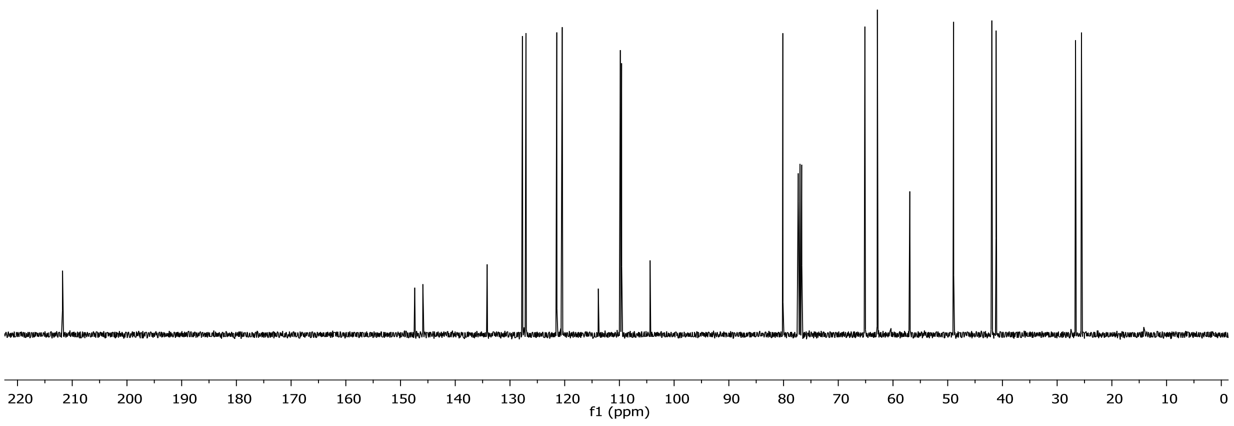

Figure S18. ^{1}H-^{1}H COSY Spectrum of Rhytidone C (**3**) in $CDCl_3$.

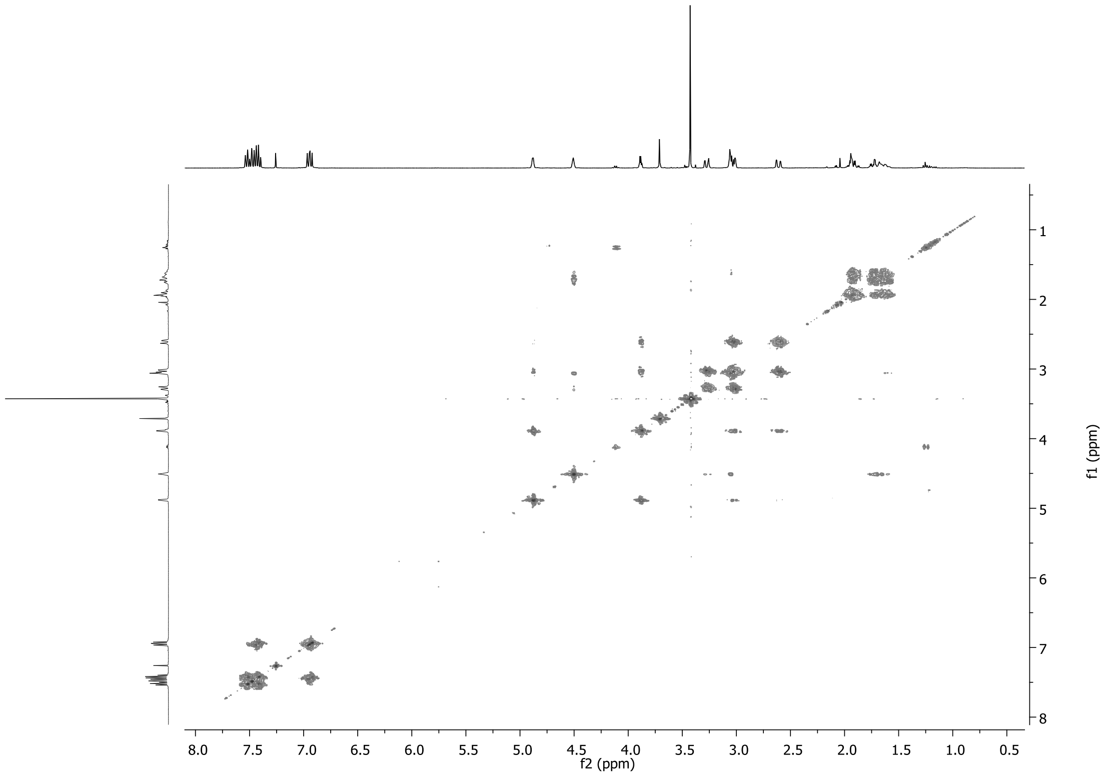

Figure S19. HSQC Spectrum of Rhytidone C (**3**) in $CDCl_3$.

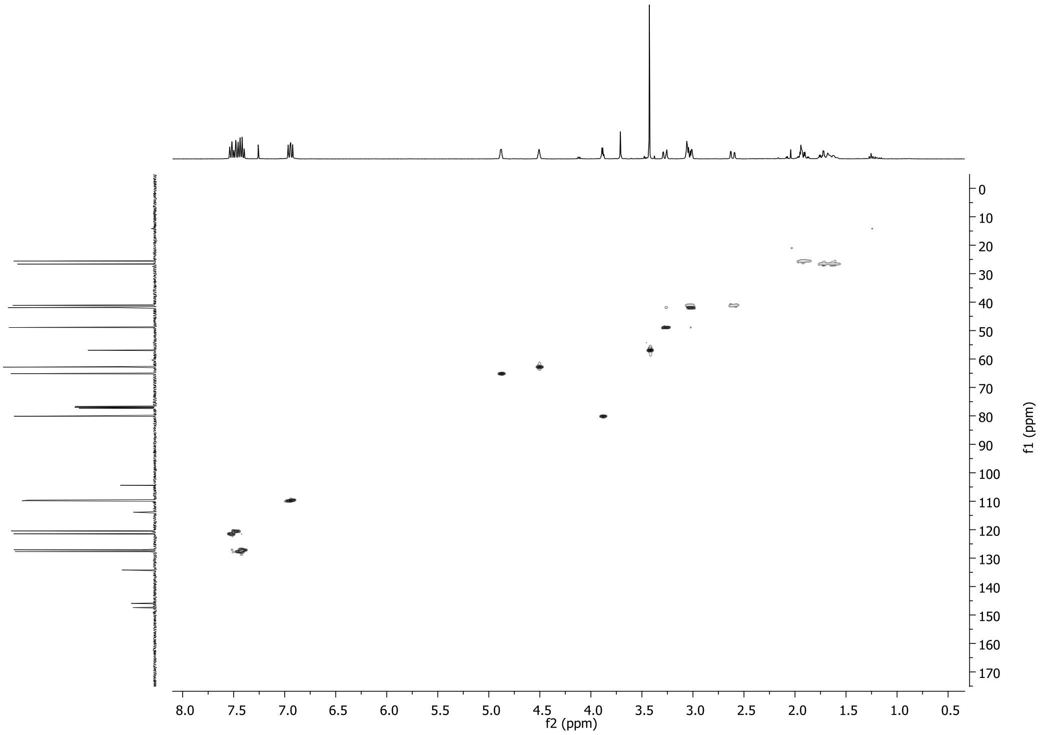

Figure S20. HMBC Spectrum of Rhytidone C (**3**) in $CDCl_3$.

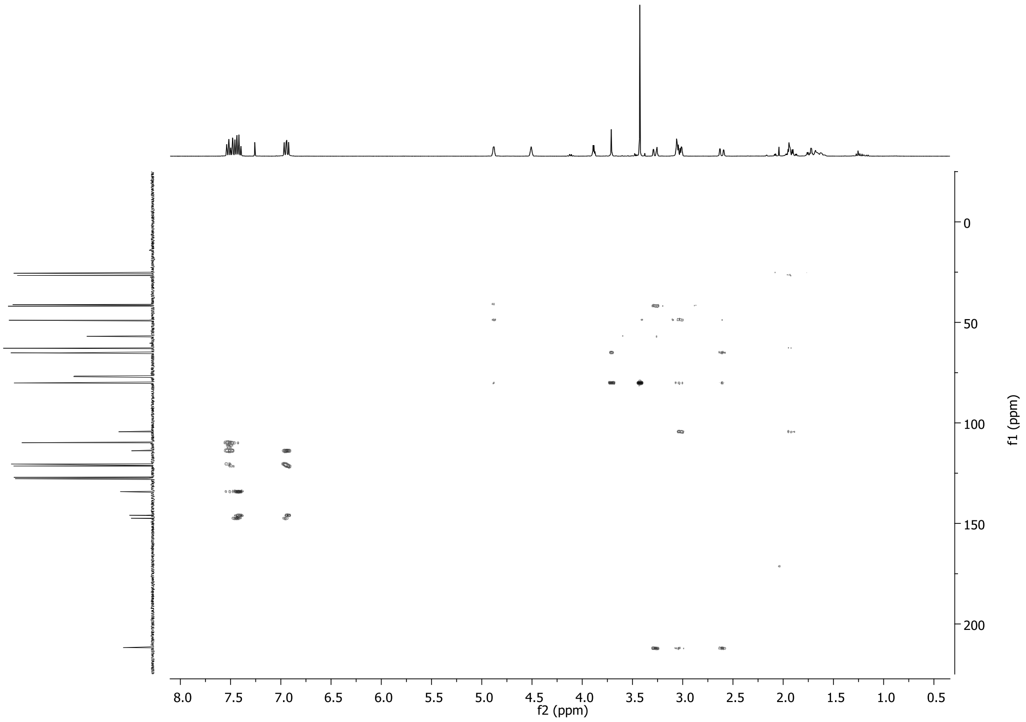

© 2014 by the authors; licensee MDPI, Basel, Switzerland. This article is an open access article distributed under the terms and conditions of the Creative Commons Attribution license (http://creativecommons.org/licenses/by/3.0/).
